# Design and testing of a soil-dividing device for a pineapple strip-tillage furrower

**DOI:** 10.1371/journal.pone.0353379

**Published:** 2026-07-17

**Authors:** Haitian Sun, Wei Zhang, Hongxuan Wang, Hailiang Li, Peng Sun, Huafen Zou, Chun Wang, Meigu Lu, Zhong Xue

**Affiliations:** 1 South Subtropical Crops Research Institute Chinese Academy of Tropical Agricultural Sciences, International Joint Research Center for the Pineapple Cultivation Techniques, Zhanjiang, China; 2 College of Engineering, Heilongjiang Bayi Agricultural University, Daqing, China; Ardakan University, IRAN, ISLAMIC REPUBLIC OF

## Abstract

To address issues such as high soil backfilling rates and difficulty in maintaining the trench shape during the operation of pineapple strip-rotary cultivators, we designed a V-shaped soil-dividing device. Structural modelling was performed using SolidWorks 2022 by establishing force analysis equations for the soil-dividing device and soil motion trajectory equations. A dynamic simulation model based on the discrete element method was constructed using the EDEM software to determine the structural parameters of the soil-dividing device. Stress analysis conducted using the ANSYS software verified that the structural strength of the soil-dividing device met the application requirements. To optimize operational parameters further, a four-factor, five-level orthogonal rotational combination field trial was designed using the central composite response surface method in Design-Expert 12. The installation distance, V-angle, blade roller speed, and forward speed were defined as the experimental factors, with the soil backfilling rate as the evaluation indicator. We analyzed the effects of various factor interactions on the soil backfilling rate. The experimental results indicated that the minimum soil backfilling rate of 38.12%, which is satisfactory for pineapple planting trenching requirements, was achieved at a V-angle of 30°, forward speed of 3.7 km/h, blade roller speed of 290 r/min, and installation distance of 125 mm. This study has significant implications for advancing the mechanization of pineapple cultivation.

## 1. Introduction

As the world’s third most important tropical fruit, pineapple cultivation quality is highly dependent on the precision and reliability of trenching operations [1]. The method and structure of trenching equipment directly influence soil disturbance levels, backfilling rates, and root development environments, which affect crop yield quality and mechanized planting efficiency [2,3]. Among the various available trenching approaches, strip-tillage furrowing demonstrates excellent potential for mechanized pineapple cultivation owing to its high operational efficiency and relatively localized soil disturbance. However, this technology currently suffers from significant shortcomings. The soil separation process is often difficult to control precisely, leading to high soil backfilling rates in trenches, poor trench stability, and uneven distribution of tilled soil on both sides of the planting trench. These issues result in insufficient soil coverage during mechanized planting. Therefore, addressing the issues of uneven soil distribution and high backfilling rates in existing strip-tillage furrowing technologies through innovative research on specialized soil-dividing devices is important for enhancing the efficiency of mechanized pineapple cultivation [4,5].

Previous research on trenching devices by scholars, both domestic and international, has primarily focused on structural innovation, numerical simulation of soil–machine interaction mechanisms, and optimization of the flow-guiding performance of soil-dividing devices. Regarding structural design, Dmytro et al. [6] proposed a combined angular trenching scheme, whereas Benjaphragairat et al. [7] emphasized the influence of the trencher shape, dimensions, and materials on performance. Ye et al.‘s [8] dual-disc corrugated structure partially improved trench depth stability. These studies predominantly addressed general agricultural scenarios without fully accounting for the specific soil coverage requirements of mechanized pineapple cultivation. Regarding numerical simulations, Ahmad and Han et al. [9,10] employed the discrete element method to analyze trenching processes, revealing the advantages of disc trenchers in terms of addressing bonding model errors and elucidating the regulatory mechanisms of spiral structure parameters on soil movement. Liu and Yang et al. [11,12] conducted orthogonal experiments and rotational speed parameter analysis, determining that while an increased rotational speed enhances soil fragmentation, it inherently leads to excessive soil ejection and reduced backfilling volume. Although such simulation studies provide reference values, their applicability to the heavy red soils prevalent in China’s primary pineapple-producing regions remains unclear. Regarding soil-dividing devices, Chen et al. [13] optimized soil movement trajectories by adding a flow-guiding mechanism. Tian et al. [14] used EDEM software simulations to confirm that distribution plates can guide soil toward the trench sides to achieve a more uniform distribution. Through orthogonal experiments, Kang et al. [15] determined that a 60° angle for the distribution plate achieved a soil coverage rate of 93.33%. However, existing research primarily focuses on crops such as wheat, maize, and medicinal plants, whose trenching requirements differ significantly from those of pineapple cultivation. Consequently, current devices struggle to meet the specific demands of pineapple planting.

In summary, current research contains gaps in the design of specialized furrowing and soil-dividing devices for pineapple cultivation, and there is a lack of structural innovation integrating red soil characteristics with planting techniques, as well as insufficient systematic optimization and field validation based on soil–machine interaction models. We address the trenching requirements of mechanized pineapple cultivation by designing a soil-dividing device for a pineapple strip-tillage furrower. Our objectives were to enhance trench quality, reduce backfilling rates, and minimize soil disturbance. Through a combination of theoretical analysis, parameter optimization, and discrete element simulation methods, we systematically investigated the structural parameters and operational performance of the proposed device. Field trials validated our findings, allowing us to provide technical support for improving trenching quality, reducing the soil backfilling rate, and advancing the mechanization of pineapple cultivation.

## 2. Materials and methods

### 2.1 Structure and working principle of the soil-dividing device

**Fig 1:**
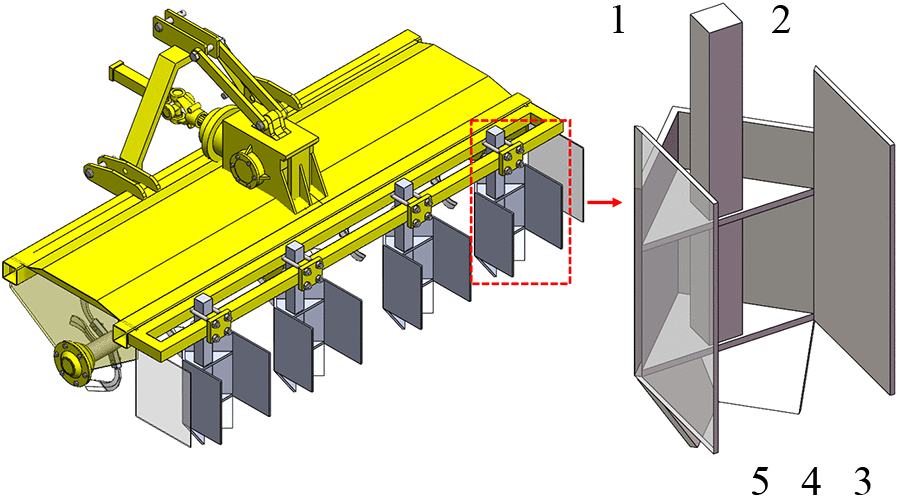
Schematic of the soil-dividing device structure. 1. connecting link 2. reinforcement plate 3. deflector plate 4. V-shaped plate 5. scraping plate.

A soil-dividing device (Fig 1) was integrated into the pineapple strip-tillage and furrowing system, consisting of a connecting link, V-shaped plate, scraping plate, deflector plate, and reinforcement plate. Its primary function is to distribute the soil that has been rotary-tilled and pulverized evenly along both sides of the seed furrow and to refine the shape of the furrow, ensuring that a standardized seed furrow is established and sufficient soil is available to cover seedlings prior to transplanting.

During operation, the tractor provides power to drive the implement forward. As the unit advances, the rotary tiller blades cut and crush the soil. The fragmented soil is ejected rearward by the blades. The V-shaped plate alters the soil particle trajectory and distributes the soil evenly on both sides of the soil-dividing device. Simultaneously, the deflector plate prevents soil backflow into the seed furrow and reduces soil refill rates. The scraping plate moves within the seed furrow and its inclined surface applies pressure on the soil against the furrow walls to achieve a standardized seed furrow shape.

### 2.2 Design and analysis of key components of the soil-dividing device

#### 2.2.1 Stress analysis and design of the V-shaped plate.

**Fig 2:**
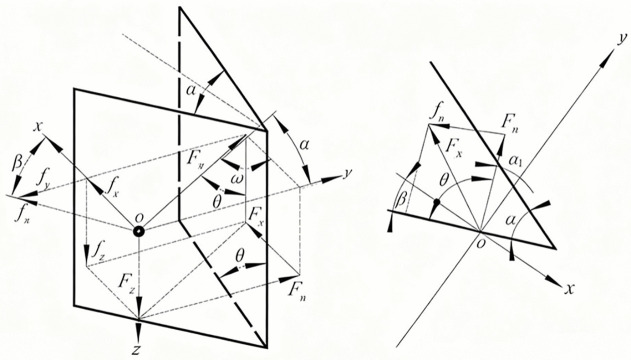
Stress analysis diagram of the V-shaped plate.

The ejection of soil fragments by a rotary tiller blade is a highly stochastic process. Randomness arises from varying soul particle sizes, initial positions on the blade, and inter-particle collisions, and soil fragments are ejected with significant randomness in terms of their trajectory and landing position. Therefore, a spatial coordinate system was established with its origin at an arbitrary point O on the contact surface of the V-shaped plate (Fig 2) to facilitate the analysis of this process. In Fig 2, the negative x axis corresponds to the negative direction of travel of the unit. The lateral normal force $F_{n}$ and sliding friction force $f_{n}$ are decomposed into vector components along the x and y axes, respectively.

According to the laws of vector projection, the magnitude of the force acting on the V-shaped plate along the z axis is zero. The relationship between $F_{n}$ and $f_{n}$ along the x axis is expressed as follows:


$$\begin{array}{c} \left\{ \begin{array}{c} @lF_{x}=F_{n}\text{cos}\omega \text{sin}\theta \\ f_{x}=f_{n}\text{cos}\beta \end{array}\right.\; \end{array}$$ (1)

where $F_{x}$ denotes the component of $F_{n}$ along the x axis (N), $f_{x}$ represents the component of $f_{n}$ along the x axis (N), ω denotes the angle between $F_{n}$ and the y axis (°), θ denotes the angle of the V-shaped plate relative to the soil (taken as 90°), and β denotes the angle between $f_{n}$ and the x axis.

Considering the stress conditions of the V-shaped plate, the following geometric relationships exist:


$$\begin{array}{c} \left\{ \begin{array}{c} @l\text{tan}\omega =\frac{\text{1}}{\text{tan}\alpha }\\ \text{cos}\beta =\text{cos}\alpha \end{array}\right.\; \end{array}$$ (2)

where α denotes the angle between the pressure exerted on the side plate and the y axis (i.e., the opening angle of the V-shaped plate (°)).

By combining Equations (1) and (2), the resultant force obstructing the advancement of the soil plate is derived as


$$\begin{array}{c} F_{f_{x}}=\frac{F_{n}\text{tan}\alpha }{\sqrt{{\text{tan}}^{\text{2}}\alpha +\text{1}}}+\frac{F_{n}\text{tan}\varphi }{\text{sin}\alpha } \end{array}$$ (3)

According to Equation (3), the resistance experienced by the V-shaped plate is influenced by the soil friction angle φ, normal pressure $F_{n}$ on the side of the V-shaped plate, and V-angle α. Under constant-operating-speed conditions, the normal force $F_{n}$ on the side of the V-shaped plate is considered constant [16]. Simulation analysis of Equation (3) using the MATLAB software revealed that the resistance force $F_{f_{x}}$ experienced by the V-shaped plate exhibits a positive correlation with α. Therefore, determining the height of the V-shaped plate alone is sufficient for establishing the structural dimensions of the soil-dividing plate.

**Fig 3:**
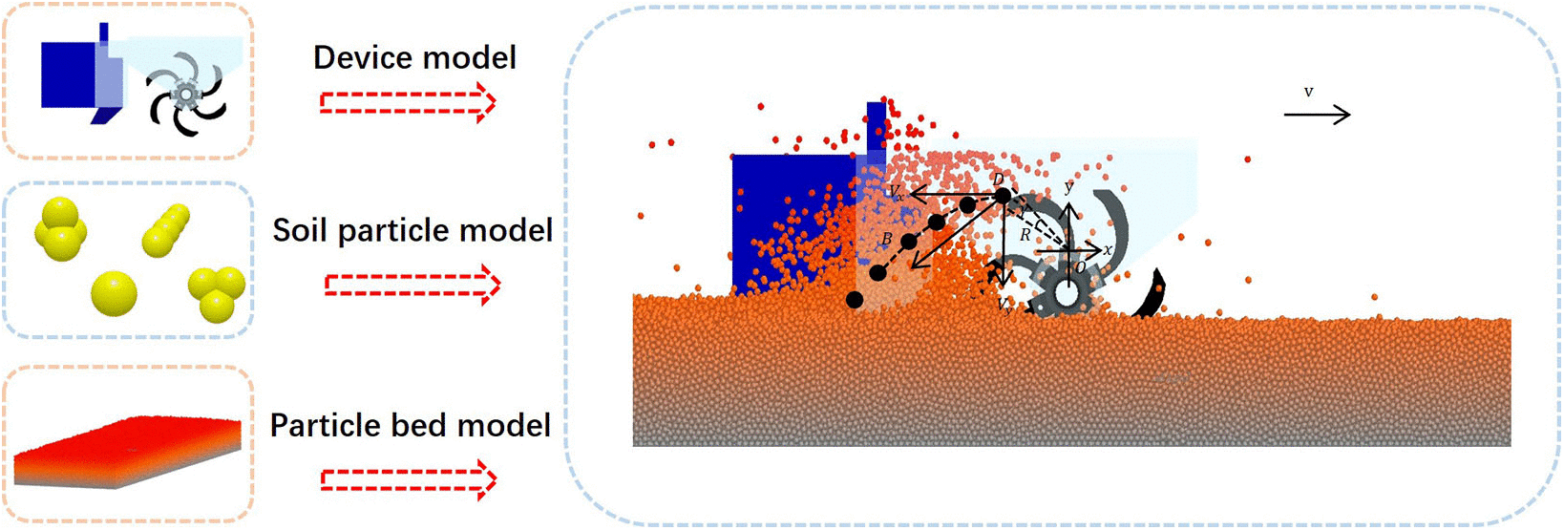
Analysis of soil particle movement during rotary tilling and furrowing.

To prevent soil particles from being ejected too high by the rotary tiller blades and passing over the V-shaped plate, the height of the V-shaped plate should exceed the maximum height reached by the soil particle movement. Accordingly, the design height of the V-shaped plate should be determined based on the maximum height achievable according to the soil movement trajectory. A small proportion of the soil is ejected rearward and upward, rebounding into the seed furrow after being deflected by the housing for secondary fragmentation. However, the majority of the soil particles are dispersed laterally and rearward along the V-shaped plate, distributing themselves on both sides of the seed furrow, as illustrated in Fig 3.

A soil particle D is considered as the subject of this analysis. Taking the line connecting the rotational center of the rotary tiller blade to the end of its tangential plane as the minimum cutting radius, and under conditions where environmental factors are disregarded, when μ > 90° [17], particle D moves along the blade’s sliding plane to the blade’s end, where it is ejected. The initial coordinates of the particle upon leaving the rotary tiller blade are as follows:


$$\begin{array}{c} \left\{ \begin{array}{c} @lx_{0}=\frac{v(\text{2}\pi -\gamma )}{\omega }+R\text{cos}\left(\text{2}\pi -\gamma \right)\\ y_{0}=R\text{sin}\left(\text{2}\pi -\gamma \right) \end{array}\right.\; \end{array}$$ (4)

where v denotes forward velocity (m/s), γ denotes tool reverse angle (rad), R denotes rotary tiller blade cutting radius (taken as 195 (mm)), $x_{0}$ denotes the initial x coordinate of particle ejection, and $y_{0}$ denotes the initial y coordinate of particle ejection.

The motion of particle *D* can be decomposed into component velocities $v_{x}$ and $v_{y}$ along the x and y axes, respectively. When ignoring environmental factors, particles follow a parabolic trajectory upon detachment from the rotary tiller blade. Therefore, the particle motion trajectory can be expressed as follows:


$$\begin{array}{c} \left\{ \begin{array}{c} @lx=x_{0}-v_{x}t\\ y=y_{0}+v_{y}t-\frac{gt^{\text{2}}}{\text{2}} \end{array}\right.\; \end{array}$$ (5)

where $v_{x}\;$ denotes the particle velocity along the negative x axis (m/s), $v_{y}$ denotes the particle velocity along the negative y axis (m/s), t denotes the time required for the particle to move from point D to point B (s), and g denotes gravitational acceleration ($m/s^{\text{2}}$).

By combining Equations (4) and (5), the particle motion height is calculated as


$$\begin{array}{c} h_{\text{2}}=R\text{sin}(\text{2}\pi -\gamma )+v_{y}t-\frac{gt^{\text{2}}}{\text{2}} \end{array}$$ (6)

According to the above equations, under conditions where the parameters R and ξ remain constant, the particle movement height h_2_ is positively correlated with its movement velocity. The calculation results indicate that when the working speed is 5 km/h, the maximum particle movement height is 327 mm.

The V-shaped plate was forged as a single piece from a 10-mm-thick 65Mn steel plate with the following structural parameters: height 330 mm, width 250 mm, angle θ between the inclined surface of the scraping plate and horizontal plane is 53°, and V-angle α provisionally set at 45°.

#### 2.2.2 Determination of the inclination angle for the scraping plate.

**Fig 4:**
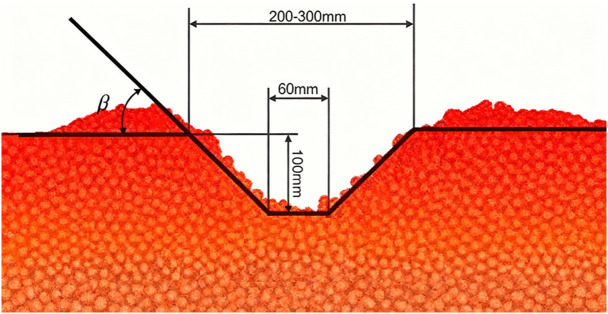
The geometric model of furrow shape.

A geometric model of the furrow shape required for mechanized pineapple cultivation is presented in Fig 4.

**Fig 5:**
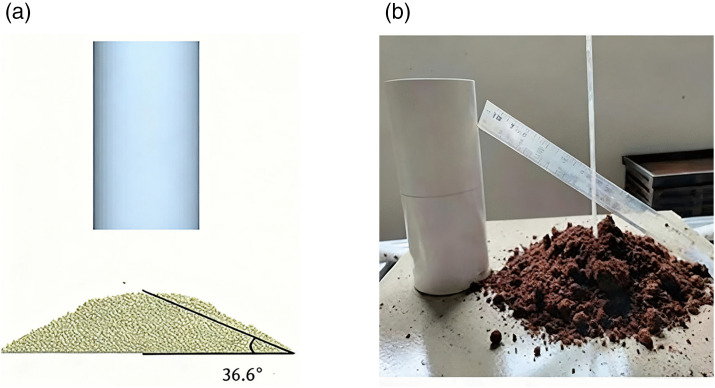
Discrete element simulation testing and physical testing. Fig (A) Simulation testing. Fig (B) Physical testing.

Fig 4 indicates that pineapple planting furrows adopt a V-shaped configuration, with $\theta^{\prime }$ representing the soil angle of repose. Consequently, we conducted discrete element calibration using the EDEM software and physical determination experiments (Fig 5) to determine the angle of repose of lateritic soils in the southern subtropical region based on the V-shaped furrow design. The EDEM calibration determined the angle of repose for lateritic soils to be 36.6°, whereas the physical measurements yielded an average angle of 37.24°. For design analysis, the angle was set to 37°.

#### 2.2.3 Stress analysis and design of the scraping plate.

**Fig 6:**
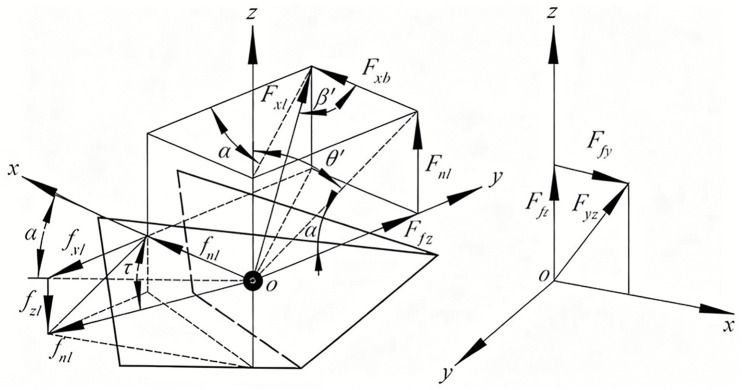
Stress analysis diagram of the scraping plate.

To investigate the influence of the soil-scraping blade on seed furrow quality, we conducted a force analysis of the interactions between the blade and soil on the furrow sidewalls. Building upon the theoretical analysis method for potato furrower–soil interactions proposed by Zhao et al. [18] we considered the regional soil characteristics of China’s southern subtropical rainforest and monsoon forest zones, namely heavy clay content, deep profile, high bulk density, pronounced swelling, plasticity, and water retention properties [19]. We focused on the interaction between normal pressure and sliding friction between the soil scraper and seed furrow sidewalls, while neglecting adhesion forces [20]. A spatial rectangular coordinate system was established at an arbitrary point O within the scraper–soil interaction (Fig 6), with the negative-x-axis direction corresponding to forward movement. During operation, the scraper plate primarily experiences a normal compressive force $F_{n\text{1}}$ (perpendicular to the inclined plate surface) and rearward sliding friction force $f_{n\text{1}}$ (parallel to the inclined plate surface) exerted by the soil, where $\theta^{\prime }$ denotes the angle of repose of the soil.

Through the theoretical analysis of the actual working process of the scraping plate, the compressive force $F_{n\text{1}}$ acting on it can be decomposed into three mutually perpendicular component forces: $F_{x\text{1}}$, $F_{y\text{1}}$, and $F_{z\text{1}}$. Based on the vector projection law and geometric relationships, the expressions for each component force are derived as follows:


$$\begin{array}{c} \left\{ \begin{array}{c} @lF_{x\text{1}}=F_{n\text{1}}\text{cos}\beta^{\prime }\\ F_{y\text{1}}=\text{sin}\beta^{\prime }\text{sin}\theta^{\prime }F_{n\text{1}}\\ F_{z\text{1}}=\text{sin}\beta^{\prime }\text{cos}\theta^{\prime }F_{n\text{1}} \end{array}\right.\; \end{array}$$ (7)

where $F_{x\text{1}}$ denotes the x component of normal pressure $F_{n\text{1}}$, $F_{y\text{1}}$ denotes the y component of normal pressure $F_{n\text{1}}$, $F_{z\text{1}}$ denotes the z component of normal pressure $F_{n\text{1}}$, $\theta^{\prime }$ denotes the angle of repose of the soil (°), and $\beta^{\prime }$ denotes the angle between normal pressures $F_{n\text{1}}$ and $F_{x\text{1}}$ (°).

Similarly, the sliding friction force $f_{n\text{1}}$ acting on the scraping plate is obtained as follows:


$$\begin{array}{c} \left\{ \begin{array}{c} @lf_{x\text{1}}=f_{n\text{1}}\text{cos}\tau \\ f_{y\text{1}}=f_{n\text{1}}\text{sin}\tau \text{sin}\beta^{\prime }\\ f_{z\text{1}}=f_{n\text{1}}\text{sin}\tau \text{cos}\beta^{\prime } \end{array}\right.\; \end{array}$$ (8)

where $f_{x\text{1}}$ denotes the component of sliding friction force $f_{n\text{1}}$ in the x direction, $f_{y\text{1}}$ denotes the component of sliding friction force $f_{n\text{1}}$ in the y direction, $f_{z\text{1}}$ denotes the component of $f_{n\text{1}}$ in the z direction, τ denotes the angle of inclination between $f_{n\text{1}}$ and the direction of travel of the unit (°), and $\beta^{\prime }$ denotes the angle between normal forces $F_{n\text{1}}$ and $F_{x\text{1}}$ (°).

By combining these geometric relationships, the following equation can be derived:


$$\begin{array}{c} \left\{ \begin{array}{c} \text{tan}\beta^{\prime }=\frac{\text{1}}{\text{tan}\alpha \text{sin}\theta^{\prime }}\\ \text{tan}\tau =\text{tan}\alpha \text{sin}\theta^{\prime } \end{array}\right.\; \end{array}$$ (9)

where α is the V-shaped plate angle (°).

Additionally, there is an identity relationship between the sliding friction and $F_{n\text{1}}$ defined as follows:


$$\begin{array}{c} f_{n\text{1}}=F_{n\text{1}}\text{tan}\varphi \end{array}$$ (10)

where φ is the friction angle between the soil and side of the V-shaped plate, which typically ranges from 15° to 38°.

By combining Equations (7)–(10) with the geometric relationship of a right-angled space, the resultant force $F_{fx\text{1}}$ acting on the x axis owing to the normal pressure and sliding friction is calculated as follows:


$$\begin{array}{c} F_{fx\text{1}}=\frac{F_{n\text{1}}(\text{tan}\alpha \text{sin}\theta^{\prime }+\text{tan}\varphi )}{\sqrt{\text{1}+{\text{tan}}^{\text{2}}\alpha {\text{sin}}^{\text{2}}\theta^{\prime }}} \end{array}$$ (11)

In summary, our analysis of the interaction between the scraping plate and soil on the seed furrow sidewall indicates that the operating resistance $F_{fx\text{1}}$ of the soil scraper increases with an increase in the V-angle α, representing a positive correlation. This result is consistent with the findings of Zhao et al. [18]. When the soil inclination angle $\theta^{\prime }$ is 37°, the operating resistance experienced by the soil scraper increases significantly with an increase in the V-angle α.

To investigate the impact of the structural parameters of the scraping plate on operational performance, discrete element single-factor simulation tests were conducted. The primary factor was the V-angle α, with evaluation metrics including the resistance force $F_{fx\text{1}}$ experienced by the scraping plate and trench width consistency coefficient (see Equation (12)). The scraping plate was designed with a vertical height of 150 mm and width of 250 mm to prevent rapid soil backfilling and ensure seed furrow stability. According to existing research, an excessively low V-angle α hinders the formation of stable V-shaped seed furrows. Therefore, the minimum value was set to 30° [21]. Simulation analyses were conducted for seven angles: 30°, 45°, 60°, 75°, 90°, 105°, and 120°.


$$\begin{array}{c} Z=1-\sqrt{\frac{\sum_{i=1}^{\text{n}}{\left(L_{i}-\overset{―}{L}\right)}^{\text{2}}}{N-1}}⬝\frac{n}{\sum_{i=1}^{n}L_{i}}\times 100\% \end{array}$$ (12)

Here, Z is the trench width consistency coefficient (%), N is the number of measurement points within a single pass, $\overset{―}{L}$ is the average trench width (mm), and n is the number of measurement points obtained per pass by the tractor.

The test soil was a lateritic red soil exhibiting pronounced expansive properties, plastic flow characteristics, and water retention capacity, with a heavy, cohesive texture. Consequently, the Johnson–Kendall–Roberts contact model was selected to describe inter-particle interactions within the soil, whereas the Hertz–Mindlin no-slip contact model was employed for interactions between the scraping plate and soil [22]. Based on preliminary experiments and a literature review, the specific simulation parameters are listed in Table 1.

**Table 1:** Simulation parameters [23,24].

Parameter	Values
Dimensions (length × width × height) (mm)	1500 × 1200 × 200
Soil density (kg·m^-3^)	2301
Soil Poisson’s ratio	0.40
Soil shear modulus (Pa)	1.12$\times {\text{1}\text{0}}^{\text{6}}$
Soil-soil kinetic friction coefficient	0.07
Soil-to-soil recovery coefficient	0.40
Soil-to-soil static friction coefficient	0.75
Density of 65Mn steel (kg·m^-3^)	7380
Poisson’s ratio of 65Mn steel	0.35
Shear modulus of 65Mn steel (Pa)	7.27$\times {\text{1}\text{0}}^{\text{1}\text{0}}$
Soil–65Mn steel recovery coefficient	0.30
Soil–65Mn steel coefficient of kinetic friction	0.05
Soil–65Mn steel static friction coefficient	0.50
Fill cell radius (mm)	8.00
Soil particle count	104030
Gravitational acceleration (m·s^-2^)	9.81
Simulation time (s)	4.30

**Fig 7:**
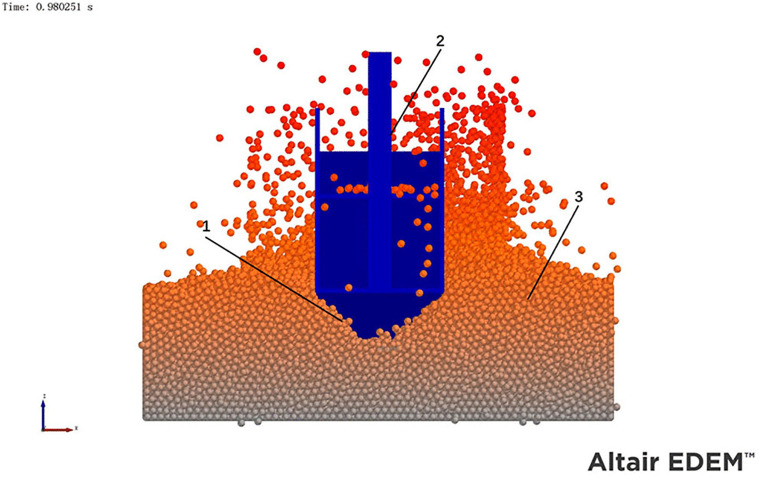
Soil simulation model. 1. scraping plate 2. connecting link 3. lateritic red soil.

Considering the operational speed range of existing supporting equipment (2–5 km/h) and the fact that the scraping plate experiences more pronounced forces at higher speeds, the simulation speed was set to 5 km/h. Models of the scraping plate and trenching device were constructed using SolidWorks 2022 and imported into EDEM 2018 in STL format for a coupled simulation with the soil particle bed, as shown in Fig 7.

The simulation process is divided into three time phases: 0–1.5 s represents the natural settling phase of soil particles with a settling velocity of –4 m/s and data recording intervals of 0.01 s, 1.5–3.0 s represents the phase of seed furrow formation by the rotary tillage furrow opener operating at a speed of 1.39 m/s, and 3.1–4.3 s represents the phase of seed furrow finishing by the scraping plate.

**Fig 8:**
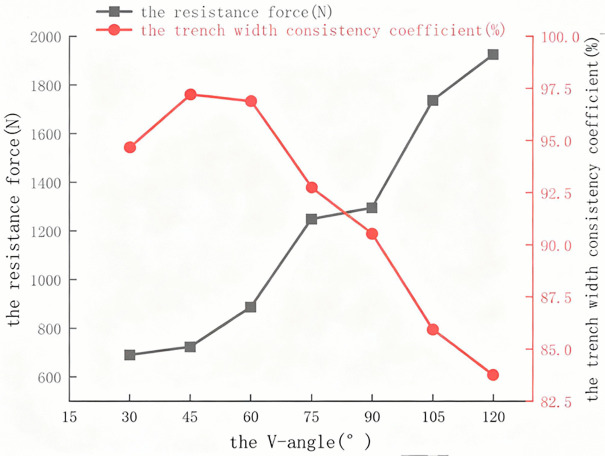
Variation curves of resistance experienced by the scraping plate and trench width consistency at different V-angle configurations.

The resistance encountered during the process from the full entry of the scraping plate into its passage through the seed furrow, along with the trench width consistency coefficient, is presented in Fig 8.

As shown in Fig 8, the V-angle α exhibits a positive correlation with the resistance force. At an angle of 30°, the resistance force is minimal at 690.6 N. Conversely, when the angle increases to 120°, the resistance force increases to 1925.27 N. This trend aligns with the results calculated using Equation (11). Furthermore, the trench width consistency coefficient attains its maximum value of 97.21% when α is 45°, with the resistance force value at this point being 690.6 N, which is relatively low. To balance structural performance and resistance characteristics, a V-angle α of 30° to 70° is deemed appropriate for the scraping plate.

### 2.3 Static stress analysis of the soil-dividing device

**Fig 9:**
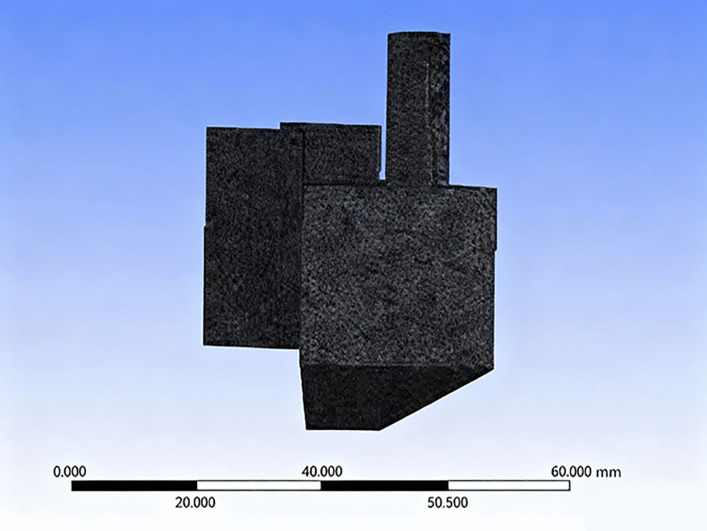
Soil-dividing device grid model.

To simulate the operational performance of the soil-dividing device accurately, a tetrahedral mesh was employed for modelling [25–27] and the corresponding material properties were defined as follows. The material was 65Mn steel with an elastic modulus of 196 GPa, yield strength of 785 MPa, and tensile strength of 980 MPa. Additional material parameters are listed in Table 1. The established mesh model consisted of 123,415 nodes and 72,225 elements, as illustrated in Fig 9.

**Fig 10:**
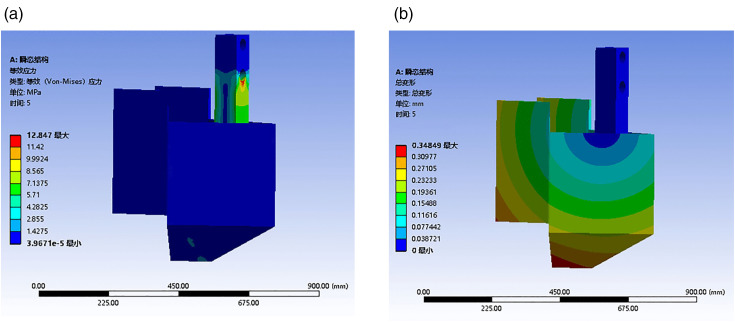
Stress–strain and total deformation contour plots. Fig (A) Stress variation. Fig (B) Total deformation.

We constrained the connecting link, applied a load of 690.6 N to the scraping plate, and set the simulation time to five seconds. The resulting stresses and total deformations are presented in Fig 10.

The soil-dividing device primarily concentrates forces on the soil-scraping plate with a maximum stress of 12.847 MPa. As the simulation time increases, the scraping plate deforms under impact forces from the complex soil structure, with a maximum displacement of 0.34849 mm. Support rods were added between the two inclined surfaces of the scraping plate to improve the load distribution. Stress analysis indicates that $\left[\sigma ]\;>\;[\sigma_{max}\right]$ [28,29], confirming that the soil-dividing device’s strength meets design requirements.

### 2.4 Field experiment

#### 2.4.1 Test conditions and factors.

**Fig 11:**
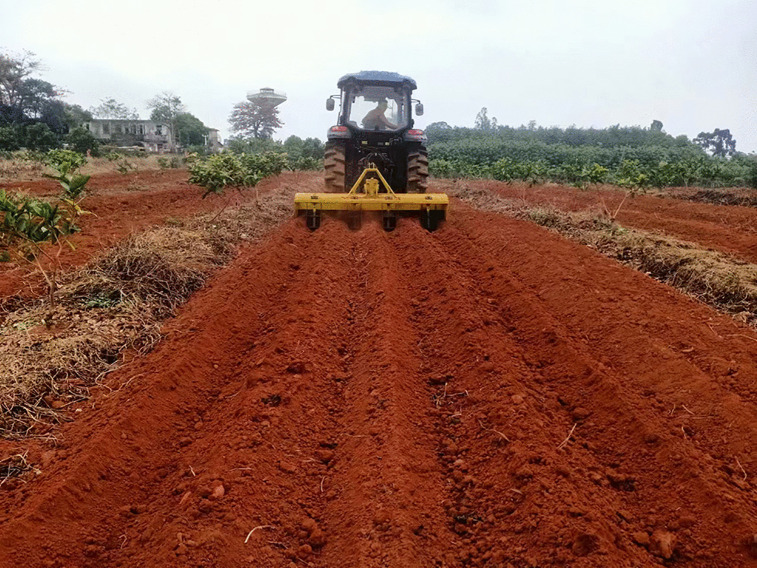
Field experiment setup.

To investigate the effects of equipment parameters on the soil backfilling rate, a field experiment was conducted, as shown in Fig 11. The experiment site was located at the National Soil Quality Zhanjiang Observation Station of the South Subtropical Crops Research Institute, Chinese Academy of Tropical Agricultural Sciences, Zhanjiang City, Guangdong Province (E109°31′, N21°35′). The test soil was southern subtropical lateritic red. The key experimental equipment included a Lovol M1004-A medium-sized tractor, strip tiller, and soil-dividing device. Based on previous theoretical analyses and simulation trials, the following factors were selected for evaluation: installation distance (distance from the soil-dividing device to the tangent of the blade roller radius of rotation), V-angle, blade roller speed, and forward speed. The soil backfilling rate was used as the evaluation metric. A four-factor, five-level orthogonal rotational combination experimental design was employed using the factor levels detailed in Table 2.

**Table 2:** Factor level table.

Level	Installation Distance *l*(mm)	Blade Roller Speed n(r/min)	Forward speed v(km/h)	V-angle α(°)
Positive Axial Distance (λ)	170	330	6	70
High Level (+1)	150	310	5	60
Center Level (0)	120	280	3.5	45
Low Level (−1)	90	250	2	30
Negative Axial Distance (−λ)	70	230	1	20

Note: λ value = 1.682, −λ value = −1.682.

#### 2.4.2 Soil backfilling rate experimental method.

The soil backfilling rate was assessed by determining the percentage of soil mass within the trench after excavation relative to the undisturbed soil mass prior to excavation within a fixed-size sampling area. The sampling area dimensions were uniformly set at 0.5 m (length) × 0.25 m (width) × 0.13 m (height). Sampling points were arranged using the standard five-point sampling method with three replicates established in each area. The results are presented as mean values. Soil samples collected before and after trenching were placed in sealed bags and weighed using a precision electronic balance. The backfilling rate was calculated as follows:


$$\begin{array}{c} F_{h}=\frac{N_{a}}{N_{b}}\times \text{1}\text{0}\text{0}\% \end{array}$$ (13)

where $F_{h}$ is the soil backfilling rate (%), $N_{a}$ is the soil particle mass in the sampling area after trenching (kg), and $N_{b}$ denotes the soil particle mass in the sampling area before trenching (kg).

## 3. Results

### 3.1 Field experiment results

A field experiment was conducted using the central composite design method in Design-Expert 12, with an installation distance $X_{\text{1}}$, blade roller speed $X_{\text{2}}$, forward speed $X_{\text{3}}$, and V-angle $X_{\text{4}}$ as experimental factors, and the soil backfilling rate as the evaluation indicator. The experimental design and results are presented in Table 3.

**Table 3:** Experimental design and results.

Trial No.	Experimental Factors				Evaluation Indicators
	Installation Distance $X_{1}$	Blade Roller Speed $X_{2}$	Forward speed $X_{3}$	V-angle $X_{4}$	Soil Backfilling Rate Y(%)
1	−1.682	0	0	0	50.26
2	−1	−1	−1	−1	45.01
3	−1	−1	−1	1	47.2
4	−1	−1	1	−1	45.48
5	−1	−1	1	1	52.44
6	−1	1	−1	−1	46.81
7	−1	1	−1	1	48.91
8	−1	1	1	−1	43.87
9	−1	1	1	1	49.84
10	0	−1.682	0	0	44.62
11	0	0	−1.682	0	48.42
12	0	0	0	−1.682	36.58
13	0	0	0	0	40.16
14	0	0	0	0	41.03
15	0	0	0	0	40.05
16	0	0	0	0	40.62
17	0	0	0	0	40.52
18	0	0	0	0	41.24
19	0	0	0	1.682	45.32
20	0	0	1.682	0	49.9
21	0	1.682	0	0	42.11
22	1	−1	−1	−1	44.55
23	1	−1	−1	1	48.39
24	1	−1	1	−1	45.81
25	1	−1	1	1	53.62
26	1	1	−1	−1	45.15
27	1	1	−1	1	47.53
28	1	1	1	−1	41.7
29	1	1	1	1	51.31
30	1.682	0	0	0	45.8

### 3.2 Regression model development and analysis of variance results

To investigate the patterns of influence exerted by variations in the experimental factors on the evaluation indicators, an analysis of variance was conducted on the test results, and the results are presented in Table 4.

**Table 4:** Analysis of variance results.

Source of Variation	Sum of Squares	Degree of Freedom	F	P
Model	491.19	14	52.08	<0.0001***
$X_{\text{1}}$	3.74	1	5.55	0.0325*
$X_{\text{2}}$	6.21	1	9.23	0.0083**
$X_{\text{3}}$	7.81	1	11.60	0.0039**
$X_{\text{4}}$	142.53	1	211.57	<0.0001***
$X_{\text{1}}X_{\text{2}}$	2.24	1	3.32	0.0885
$X_{\text{1}}X_{\text{3}}$	0.6084	1	0.9031	0.3570
$X_{\text{1}}X_{\text{4}}$	2.58	1	3.82	0.0694
$X_{\text{2}}X_{\text{3}}$	12.04	1	17.87	0.0007***
$X_{\text{2}}X_{\text{4}}$	0.0342	1	0.0508	0.8247
$X_{\text{3}}X_{\text{4}}$	24.60	1	36.52	<0.0001***
$\overset{\text{2}}{\underset{\text{1}}{X}}$	112.65	1	167.22	<0.0001***
$\overset{\text{2}}{\underset{\text{2}}{X}}$	15.58	1	23.12	0.0002***
$\overset{\text{2}}{\underset{\text{3}}{X}}$	149.54	1	221.98	<0.0001***
$\overset{\text{2}}{\underset{\text{4}}{X}}$	0.2451	1	0.3639	0.5554
Residual	10.11	15		
Lack of fit	9.01	10	4.10	0.0663
Error	1.10	5		
Total	501.30	29		

Note: “***” indicates extremely significant (P<0.001), “**” indicates highly significant (p< 0.01), “*” indicates significant (0.01 <p< 0.05), and *P* > 0.05 indicates insignificant.

Table 4 reveals that the regression model for the soil backfilling rate Y was extremely significant (P < 0.0001), whereas the lack of fit was insignificant (P > 0.05). These results confirm the excellent fit of the model, rendering the regression equation practically meaningful. The effects of blade roller speed $\left(X_{2}\right)$ and forward speed $\left(X_{3}\right)$ are highly significant (P < 0.01), while the linear term of installation distance $\left(X_{1}\right)$ reaches a significant level. The interaction terms $X_{1}X_{2},\;X_{1}X_{3},\;X_{1}X_{4},\;and\;X_{2}X_{4}$, as well as the quadratic term $\overset{2}{\underset{4}{X}}$, are all insignificant (P > 0.05), whereas the remaining terms are extremely significant (P < 0.0001). The order of influence of the experimental factors on the evaluation indicator is as follows: V-angle $\left(X_{4}\right)$> forward speed $\left(X_{3}\right)$> blade roller speed $\left(X_{\text{2}}\right)$> installation distance $\left(X_{1}\right)$. The results indicate that the soil backfilling rate exhibits a quadratic relationship with the interaction effects among the experimental factors, rather than a simple linear relationship [30–32]. After eliminating insignificant terms, the regression equation is written as follows:


$$\begin{array}{c} Y=\text{4}\text{0}.\text{6}\text{0}-\text{0}.\text{4}\text{1}X_{\text{1}}-\text{0}.\text{5}\text{3}X_{\text{2}}+\text{0}.\text{6}\text{0}X_{\text{3}}+\text{2}.\text{5}\text{7}X_{\text{4}}-\text{0}.\text{8}\text{6}X_{\text{2}}X_{\text{3}}+\text{1}.\text{2}\text{4}X_{\text{3}}X_{\text{4}}+\text{2}.\text{2}\text{6}\overset{\text{2}}{\underset{\text{1}}{X}}+\text{0}.\text{9}\text{8}\overset{\text{2}}{\underset{\text{2}}{X}}+\text{3}.\text{0}\text{2}\overset{\text{2}}{\underset{\text{3}}{X}} \end{array}$$ (14)

### 3.3 Response surface analysis

**Fig 12:**
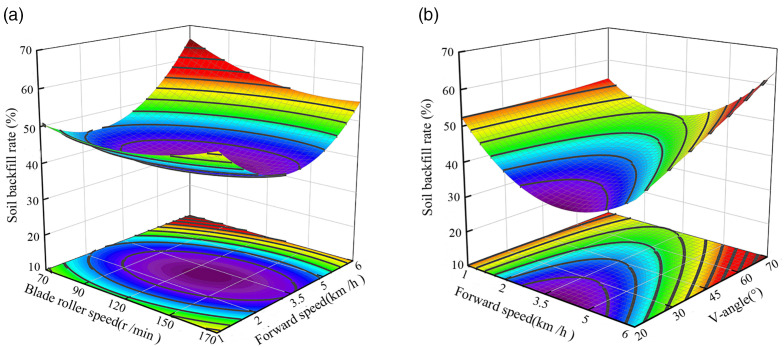
Interaction effects on evaluation indicators. Fig (A) Interaction between blade roller speed and forward speed. Fig (B) Interaction between V-angle and forward speed.

As shown in Fig 12a, the soil backfilling rate is subject to an extremely significant interaction between the blade roller speed and forward speed. When the blade roller speed remains constant, the soil backfilling rate first decreases and then increases with forward speed. At low speeds, the extended contact time of the rotary tiller blades with the seed furrow area leads to excessive tilling and significant soil ejection beyond the furrow, reducing the backfilling volume. At medium-to-high speeds, the reduced number of cuts per unit distance increases the proportion of soil falls back into the furrow, causing the backfilling rate to increase. At a constant forward speed, the backfilling rate also exhibits a non-monotonic change with increasing blade roller speed, first decreasing and then increasing. Within the low-to-medium speed range, higher speeds facilitate more thorough soil cutting and efficient transport by rotary blades to the rear soil-dividing device, promoting a more uniform soil distribution on both sides of the seed furrow, thereby reducing the backfilling rate. However, when the blade roller speed exceeds a critical threshold, the centrifugal force generated by the blade roller increases significantly, causing the soil particles to scatter violently and randomly, thereby weakening their directional transport capability. Consequently, a large amount of soil falls back into the furrow, causing the backfilling rate to increase.

As shown in Fig 12b, there is an extremely significant interaction between the forward speed and V-angle α impacting soil backfilling efficiency. At a constant forward speed, the backfilling rate increases significantly as the V-angle increases. This trend can be explained as follows. A larger V-angle diminishes the lateral soil-cutting capability and reduces the efficiency of directed soil transport along the plate surface. Simultaneously, an increased angle exacerbates the fallback of soil particles into the seed furrow, causing more soil to re-enter the furrow, thereby increasing the backfilling rate.

### 3.4 Parameter optimization

To minimize the soil backfilling rate, the nonlinear optimization theory was employed to optimize the established regression model while considering the experimental constraints. The constraints and objective functions are defined as follows:


$$\begin{array}{c} F=\left\{ \begin{array}{c} @lminY=(X_{\text{1}},X_{\text{2}},X_{\text{3}},X_{\text{4}})\\ s.t.\left\{ \begin{array}{c} @l\text{7}\text{0}mm\leq X_{\text{1}}\leq \text{1}\text{7}\text{0}mm\\ \begin{array}{c} @l\text{2}\text{3}\text{0}r/min\leq X_{\text{2}}\leq \text{3}\text{3}\text{0}r/min\\ \begin{array}{c} @l\text{1}km/h\leq X_{\text{3}}\leq \text{6}km/h\\ \text{2}\text{0}\circ \leq X_{\text{4}}\leq \text{7}\text{0}\circ \end{array} \end{array} \end{array}\right.\; \end{array}\right.\; \end{array}$$ (15)

Using Design-Expert 12 to optimize the regression model yielded the following results. When the installation distance (X_1_) was 125 mm, blade roller speed (X_2_) was 290 r/min, forward speed (X_3_) was 3.7 km/h, and V-angle (X_4_) was 30°, the soil backfilling rate was minimized at 38.12%.

### 3.5 Verification experiment

To validate the accuracy of the test results and determine the optimal operating parameters for the soil-dividing device, tests were conducted using the optimized combination of parameters. Five test groups were established, each repeated thrice, and average values were calculated. The results are summarized in Table 5.

**Table 5:** Optimal parameter validation results.

Group	Soil Backfilling Rate (%)
1	42.55
2	40.64
3	36.90
4	45.84
5	37.17
Average	40.62

As shown in Table 5, during the validation tests employing the optimized parameters, the average soil backfilling rate was 40.62%, which is consistent with the previously determined optimal value (38.12%). This result indicates that the established optimal working parameter combination is stable and reliable.

## 4. Discussion

The V-plate angle α is crucial for balancing the guiding efficiency and operational resistance of the soil-dividing device. Our analysis revealed that as the V-angle increases from 30° to 120°, the device’s forward resistance linearly increases from 690.6 N to 1925.27 N, while the soil backfilling rate also significantly increases (Fig 8). This phenomenon primarily stems from two physical mechanisms. First, an enlarged V-angle weakens the lateral pressure component ($F_{x}$) exerted by the soil on the plate wall, reducing the lateral guiding capability of the V-shaped plate. Additionally, a large angle shortens the effective path for soil particles to move toward the trench side, causing numerous particles to fall back into the planting trench as a result of insufficient guidance by inertia. Second, a larger V-angle concentrates the soil–plate contact stress distribution, intensifying soil particle aggregation and further increasing the probability of particle rebound.

When the V-angle was 30°, the device exhibited optimal lateral guidance capabilities, ensuring that the soil particles were thoroughly dispersed toward the trench sides. Concurrently, the operational resistance was minimized under these conditions, effectively preventing equipment vibrations caused by a resistance overload. ANSYS static stress analysis results substantiate this finding. The maximum equivalent stress (12.847 MPa) remains substantially below the yield strength of 65Mn steel (785 MPa), whereas the maximum deformation (0.34849 mm) fully satisfies the structural strength and operational stability design requirements [28,29]. The trend in the trench width consistency coefficient further confirms that selecting a smaller V-angle maintains over 95% trench width consistency while controlling the backfilling rate to a low level, achieving an optimal balance between soil separation efficiency, trench shape stability, and operational resistance.

Based on the above analysis, a V-angle range of 30°–70° represents the optimal interval for achieving the aforementioned three-dimensional equilibrium. This conclusion aligns closely with theoretical analysis based on soil particle trajectories (Equations (5) and (6)). Within this range, the 330 mm design height of the V-shaped plate is sufficient to intercept the maximum ejection height of 327 mm for laterite particles completely, effectively preventing the occurrence of particles bypassing the plate and returning to the soil at their source.

The influence of the operational parameters on soil separation was equally pronounced. An analysis of variance indicated that the interaction between blade roller speed ($X_{2}$) and forward speed ($X_{3}$) exerts a highly significant effect on the soil backfilling rate (P < 0.0001). The underlying regulatory mechanism lies in the dynamic equilibrium between the adequacy of soil cutting and efficiency of particle orientation. When the forward speed falls below 3.7 km/h, the contact time between the blade roller and soil increases, enhancing soil fragmentation. The centrifugal force generated by the blade roller efficiently propels the fragmented soil toward the V-shaped plate, improving particle guidance toward the trench sides and consequently reducing the backfilling rate. Conversely, at speeds exceeding 3.7 km/h, insufficient soil cutting causes unbroken aggregates to fall vertically under gravity, leading to increased backfilling rates. The rotational speed of the blade roller exhibits a threshold effect: within the range of 230–290 r/min, increasing the speed enhances the centrifugal force, promotes particle guidance, and reduces the backfilling rate. However, when exceeding 290 r/min, excessive centrifugal force causes particle trajectories to deviate from the guidance range of the V-shaped plate, resulting in random scattering and reversing the reduction in the backfilling rate.

To address the unique challenges of red soils in thermal zones, which are characterized by high clay content, strong expansivity, and a tendency to agglomerate [19,24], we derived a deep coupling between the soil-dividing device structure and both soil properties and agronomic requirements. This coupling revealed optimal parameter ranges, markedly contrasting with prior research findings on other soil types. The innovation of our study lies in the systematic establishment of the relationships between soil physicochemical properties, structural parameters, and operational performance. The key physical parameters of red soil (angle of repose = 37°, density = 2301 kg/m³, Poisson’s ratio = 0.4) were calibrated using the EDEM discrete element simulation software. The Johnson–Kendall–Roberts contact model was employed to simulate particle cohesion in red soil precisely [22], thereby overcoming the significant simulation deviations observed in prior studies. Concurrently, the core structural parameters were optimized. The 330 mm height of the V-shaped plate precisely intercepts particle ejection, while the 53° scraping blade inclination aligns with the red soil angle of repose. Moderate compaction of the trench walls ensures a consistent trench width, further reducing soil return sources. Field validation demonstrated that our coupled design reduces red soil backfilling rates by 12% to 22% compared with conventional soil-dividing plates. Our soil-dividing device represents the first systematic structural solution for pineapple strip-tillage trenching equipment under red soil conditions in thermal zones, effectively overcoming the technical bottlenecks of incomplete soil division and persistently high backfilling rates encountered by traditional equipment in red soils.

The installation distance refers to the horizontal distance between the soil-dividing device and the tangent line of the blade roller’s rotation circle, which is a key structural parameter affecting soil movement trajectories and backfilling performance. Combined with the particle motion Equations (4) and (5), the installation distance directly changes the initial relative position between ejected soil particles and the V-shaped plate. When the installation distance is small, soil particles thrown by rotary blades strike the V-shaped plate at a shorter flight distance. The particles retain high kinetic energy after collision, which enhances the lateral diversion effect and effectively prevents soil from falling back into the trench. As the installation distance increases, the flight path of soil particles lengthens, and the particle velocity gradually attenuates before contacting the soil-dividing device. Reduced kinetic energy weakens the lateral guiding capacity of the V-shaped plate, and more soil particles fail to be transported to both sides of the trench, resulting in a higher soil backfilling rate.

According to the regression model and experimental results, the installation distance has a significant effect on the soil backfilling rate. Within the test range of 70–170 mm, the backfilling rate first decreases and then increases with the rise of installation distance. At the optimal installation distance of 125 mm, the soil particles fully interact with the V-shaped plate, achieving the best soil separation effect and the lowest backfilling rate. Excessively large or small installation distances will break the matching relationship between particle motion trajectory and the soil-dividing structure, and deteriorate the overall working performance of the device.

In summary, we significantly reduced the soil backfilling rate of pineapple strip-tillage furrow openers under red soil conditions by optimizing the key parameters in a V-shaped soil-dividing device. The core value of this study lies in the pioneering of a systematic solution based on soil–machine dynamic mechanisms, specifically for tropical crops and red soils, filling a gap in the existing research. Our findings not only provide direct technical support for mechanized pineapple cultivation but also offer novel insights for innovative agricultural equipment design under specialized soil conditions. Subsequent research will focus on exploring the multi-soil adaptability, long-term durability, and combined operational efficiency of the proposed device.

## 5. Conclusions

In this study, we designed and analyzed a soil-dividing device for pineapple strip-tillage trenching equipment. Through a combined approach based on simulations and field experiments, the structural and operational parameters of the proposed device were optimized and validated. The following conclusions were drawn.

(1) Theoretical analysis of the soil-dividing device revealed that the resistance force experienced by the V-shaped plate and soil-scraping plate during operation increases significantly as the V-angle α increases. Furthermore, the trench width consistency coefficient exhibits a nonlinear trend with an increasing angle α, first rising and then decreasing, providing a theoretical basis for selecting the key operating parameters of the soil-dividing device.
(2) Through discrete element simulations and analysis, the optimal parameters for the core structure of the soil-dividing device were determined. The optimal V-shaped plate height is 330 mm with a width of 250 mm. The angle θ between the inclined surface of the scraping plate and horizontal plane should be 53°.
(3) Based on an analysis of variance and model parameters, the factors affecting the peak force, in order of significance, were determined to include the V-angle α, forward speed, blade roller speed, and installation distance. Regarding interactions, the forward speed and blade roller speed, and the forward speed and V-angle α exert extremely significant coupled influences on the soil backfilling rate.
(4) Based on field experiment results, the optimal parameter combination for the soil-dividing device was determined. When the installation distance ($X_{1}$) is 125 mm, blade roller speed ($X_{2}$) is 290 r/min, forward speed ($X_{3}$) is 3.7 km/h, and V-angle ($X_{4}$) is 30°, the soil backfilling rate is minimized to only 38.12%. This parameter combination significantly enhances the quality of trenching operations, providing a basis for optimizing the design of soil-dividing devices and selecting field operation parameters.

## Supporting information

S1 Data Data. (XLSX)
